# Interferon-alpha and angiogenic dysregulation in pregnant lupus patients destined for preeclampsia

**DOI:** 10.1186/ar4644

**Published:** 2014-09-18

**Authors:** Danieli Andrade, Mimi Kim, Luz P Blanco, S Ananth Karumanchi, Gloria C Koo, Patricia Redecha, Kyriakos Kirou, Angela M Alvarez, Melissa J Mulla, Mary K Crow, Vikki M Abrahams, Mariana J Kaplan, Jane E Salmon

**Affiliations:** 1 Hospital for Special Surgery and Weill Cornell Medical College, New York, NY, USA; 2 Albert Einstein College of Medicine of Yeshiva University, Bronx, NY, USA; 3 Systemic Autoimmunity Branch, Intramural Research Program, National Institute of Arthritis and Musculoskeletal and Skin Diseases, National Institutes of Health, Bethesda, MD, USA; 4 Beth Israel Deaconess Medical Center, Harvard Medical School and the Howard Hughes Institute, Boston, MA, USA; 5 University of Antioquia, School of Medicine, Medellin, Colombia; 6 Yale School of Medicine, New Haven, CT, USA

## Background

Pregnant patients with SLE are at increased risk of placental insufficiency and preeclampsia, disorders associated with angiogenic factor imbalance. IFNα, a critical element in SLE pathogenesis, is a potent antiangiogenic factor. In a case-control longitudinal study of lupus pregnancies from PROMISSE (Predictors of Pregnancy Outcome: Biomarkers In Antiphospholipid Antibody Syndrome and Systemic Lupus Erythematosus), we investigated whether elevated IFNα early in pregnancy might be associated with poor pregnancy outcomes.

## Methods

Each of 28 SLE patients with poor pregnancy outcome was matched to an SLE patient with an uncomplicated pregnancy and to a pregnant healthy control. Serum samples obtained monthly through pregnancy were assayed for IFNα activity using a reporter cell assay, and for antiangiogenic factor, sFlt1, and proangiogenic factor, placenta growth factor (PlGF). Human umbilical vein endothelial cells (HUVEC) were cultured in the presence of IFNα and/or sFlt1, and gene expression assessed by q-RT PCR. The effect of IFNα and sFlt1 on endothelial-trophoblast interactions was assessed in an *in vitro *model of spiral artery transformation in which the capacity of human first trimester extravillous trophoblasts to stabilize endometrial endothelial cell tube structures is measured.

## Results

Compared with SLE patients with uncomplicated pregnancies, those with preeclampsia had increased IFNα before clinical symptoms. Nonautoimmune patients destined for preeclampsia did not have increased IFNα. In SLE patients with low IFNα, marked angiogenic imbalance (higher sFlt1, lower PlGF and higher sFlt1/PLGF ratios) precedes maternal manifestations of preeclampsia, whereas in SLE with high IFNα, preeclampsia occurs without evidence of systemic angiogenic imbalance. To investigate this result, we treated HUVEC with exogenous sFlt1 and IFNα. Treatment with sFlt1 induced the expression of *sFlt1 *mRNA, and IFNα dramatically amplified the endothelial response to sFlt1, leading to a local positive feedback loop. Furthermore, in an *in vitro *model of spiral artery transformation, only IFNα and sFlt1 together disrupted the ability of trophoblast cells to remodel endothelial tube structures.

## Conclusions

Our studies identify a new mechanism by which IFNα induces an antiangiogenic milieu in the vasculature leading to inadequate spiral artery remodeling and poor placentation early in pregnancy and maternal endothelial dysfunction presenting as preeclampsia later in pregnancy. They suggest that elevated IFNα may contribute to the pathogenesis of preeclampsia in some pregnant women with SLE.

